# Meta-Analysis and Evidence Base for the Efficacy of Autologous Bone Marrow Mesenchymal Stem Cells in Knee Cartilage Repair: Methodological Guidelines and Quality Assessment

**DOI:** 10.1155/2019/3826054

**Published:** 2019-04-07

**Authors:** Mohamed E. Awad, Khaled A. Hussein, Inas Helwa, Mohamed F. Abdelsamid, Alexandra Aguilar-Perez, Ibrahim Mohsen, Monte Hunter, Mark W. Hamrick, Carlos M. Isales, Mohammed Elsalanty, William D. Hill, Sadanand Fulzele

**Affiliations:** ^1^Micro-CT Core, Oral Biology Department-Augusta University, Augusta, GA, USA; ^2^Oral and Maxillofacial Surgery-Oral and Dental Research Division-National Research Centre, Cairo, Egypt; ^3^Faculty of Oral and Dental Medicine, Misr International University, Cairo, Egypt; ^4^Department of Surgery, Mayo Clinic, Rochester, MN, USA; ^5^Anatomy and Cell Biology Department-Indiana University School of Medicine, Indianapolis, IN, USA; ^6^Department of Orthopaedic Surgery, Fayoum University, Fayoum, Egypt; ^7^Department of Orthopaedic Surgery, Augusta University, Augusta, GA, USA; ^8^Institute of Regenerative and Reparative Medicine, Augusta University, Augusta, GA, USA; ^9^Cell Biology and Anatomy Department, Augusta University, Augusta, GA, USA; ^10^Division of Endocrinology, Diabetes and Metabolism, Medical College of Georgia, Augusta University, Augusta, GA 30912, USA; ^11^Department of Pathology and Laboratory Medicine, Medical University of South Carolina, Charleston, SC 29403, USA

## Abstract

The aim of this study is to review all the published clinical trials on autologous bone marrow mesenchymal stem cells (BM-MSCs) in the repair of cartilage lesions of the knee. We performed a comprehensive search in three electronic databases: PubMed, Medline via Ovid, and Web of Science. A systematic review was conducted according to the guidelines of PRISMA protocol and the Cochrane Handbook for Systematic Reviews of Interventions. The modified Coleman methodology score was used to assess the quality of the included studies. Meta-analysis was conducted to estimate the effect size for Pain and function change after receiving BM-MSCs. Thirty-three studies—including 724 patients of mean age 44.2 years—were eligible. 50.7% of the included patients received cultured BM-MSCs for knee cartilage repair. There was improvement in the MINORS quality score over time with a positive correlation with the publication year. Meta-analysis indicated better improvement and statistical significance in the Visual Analog Scale for Pain, IKDC Function, Tegner Activity Scale, and Lysholm Knee Score after administration of noncultured BM-MSCs when compared to evaluation before the treatment. Meanwhile, there was a clear methodological defect in most studies with an average modified Coleman methodology score (MCMS) of 55. BM-MSCs revealed a clinically relevant improvement in pain, function, and histological regeneration.

## 1. Introduction

Cartilage degeneration is the main cause of knee joint altered function and flexibility [1]. Regardless of the cause of cartilage degeneration (aging, obesity, trauma, repetitive overuse, or arthritis), spontaneous healing of full-thickness articular cartilage lesions is unlikely due to the limited regenerative capacity of chondrocytes [2]. If left untreated, full-thickness cartilage defects would ultimately increase the risk of subsequent osteoarthritis (OA) with severe associated pain and limited mobility [3]. Knee OA accounts for 80% of the total OA population based on the Global Burden of Disease (GDB) study with health care costs substantially rising every year [4]. According to the Medical Expenditure Panel Survey (MEPS), between years 1995 and 2005, the US annual expenditure estimates of out-of-pocket costs, health insurance costs, and total direct costs secondary to osteoarthritis were $185.5 billion [5].

The management of cartilage degeneration has been established over the consequent decades. The therapeutic options range from conservative therapy to restorative approaches. These treatment options have been developed to alleviate symptoms (mainly pain) and improve function and mobility, in addition to limiting the lesion progression. Conservative therapy includes either physiotherapy measures or pharmacological agents such as chondroprotective agents (e.g., D-glucosamine sulfate, chondroitin sulfate, and diacerein) and intra-articular injection of hyaluronic acid [6, 7]. Unfortunately, none of these agents could repair and heal the cartilage defects; it only might alleviate the associated pain and partially improve the knee function [8]. Several surgical approaches have been performed for cartilage repair, either reparative approaches such as microfracture [9] and abrasion or restorative approaches such as autologous chondrocyte implantation (ACI) [10], osteochondral cartilage autograft transfer (OAT) [11], or osteochondral allograft transplantation (OCA) [12]. In addition, total knee replacement is considered as the only therapeutic option for severe knee osteoarthritis [13] .

The mechanical flexibility of the knee joint requires preserved articular cartilage; this preservation also is highly dependent on both the level of single cells and chondrocytes and the whole organized tissue architecture [14]. Over the last ten years, cellular-based therapy and tissue engineering approaches have been used to repair knee cartilage defects. The subchondral progenitor cell population diminished with age [15]; the administration of autologous bone marrow mesenchymal stem cells (BM-MSCs) is considered a new promising alternative therapy for articular cartilage regeneration (stage if possible). The self-renewal capability of BM-MSCs, their potential to differentiate into chondrocytes, and their limited immunogenicity, in addition to their easy accessibility from multiple sources, highlight the feasibility of using BM-MSCs with promising potential in regeneration of knee cartilage defects [16–18] .

Different approaches are currently performed to use autologous BM-MSCs in knee cartilage repair. Direct intra-articular injection of autologous MSCs either cultured or noncultured is considered as a technically simple approach to deliver the cells into cartilage defects. Another interesting approach especially for full-thickness defect is scaffold-cell composite to restore the whole structural and biomechanical characteristics of articular cartilage [19]. Eventually, there is serious necessity to clarify the performed procedures and approaches in cellular manipulation to achieve the optimal functional and histological outcomes and determine whether the use of BM-MSCs is safe, feasible, and effective in knee cartilage regeneration. To the best of our knowledge, there are no definite guidelines for this novel therapeutic trend. Therefore, the aim of this systematic review is to analyze the methodology and outcome data of the clinical trials which used BM-MSCs as a curative option for knee articular cartilage degeneration. In addition, our systematic review and meta-analysis may aid to set up the recommendations for guidelines for this new treatment modality.

## 2. Materials and Methods: Protocol and Registration

We systematically reviewed all clinical studies investigating the use of bone marrow MSCs in knee cartilage repair. This review follows the PRISMA (Preferred Reporting Items for Systematic Reviews and Meta-Analyses) Statement and implemented the quality checklist as mentioned in the Cochrane Reviewers' Handbook [20].

### 2.1. Search Strategy

We systematically searched for all relevant articles in 3 online databases, PubMed, MEDLINE via Ovid, and Web of Science, according to the instructions of the “optimum trial search strategy” described in the Cochrane Reviewers' Handbook. To ensure the inclusion of all relevant studies, we manually retrieved any related clinical studies in the references of the included studies. The Boolean operations and keywords used for the search were “bone marrow aspirate concentrate” OR “bone marrow aspirate” AND “knee.” The terms also were crossed as follows: exp Cartilage, Articular/OR exp Osteochondritis/OR osteochondral^∗^.mp. OR exp Knee Joint. We extended the inclusion criteria to studies that were cohort trials, case series, and case reports if the treatment group received MSCs and conducted in appropriate clinical settings. In vitro and in vivo investigations that used animal experiments were excluded from the analysis. In addition, we also reviewed the unpublished clinical trials via clinicalTrials.gov to be drafted.

### 2.2. Study Selection

**Figure 1:**
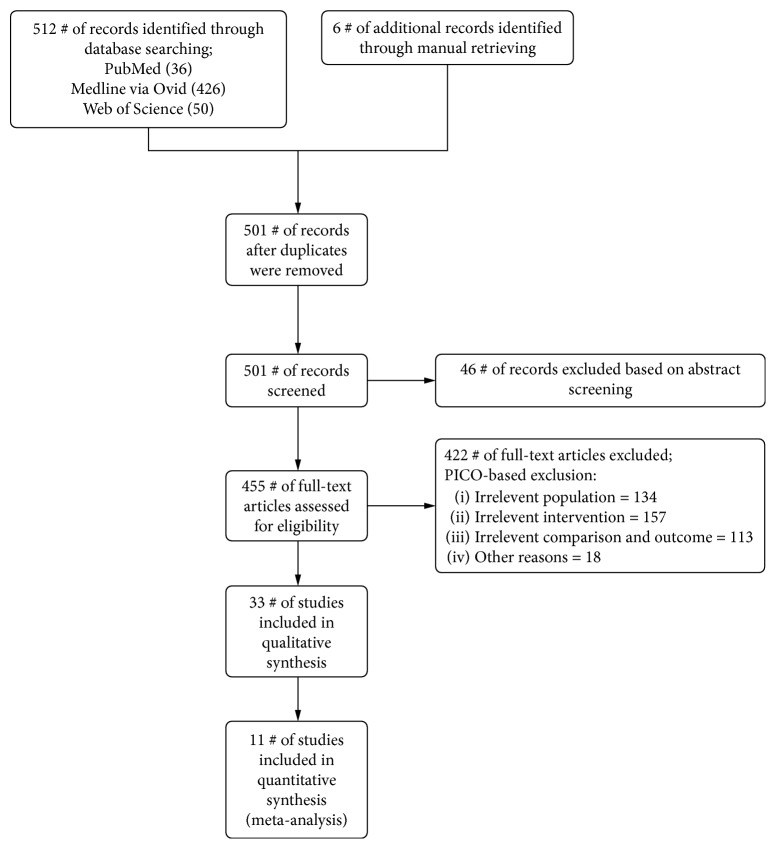
Flow chart showing search strategy and study identification, inclusion, and exclusion.

Inclusion criteria were applied on all full-text articles to select the English language, human clinical trials investigating the use of bone marrow aspirate for the repair of cartilage defects of the knee. Studies that did not match the inclusion criteria were excluded, and exclusion reasons were noted. The process of study identification is presented in Figure 1.

### 2.3. Data Collection and Data Items

Two review authors extracted data independently from the included studies with a self-designed table. Contents of the data extraction included type of study design, level of evidence, method of delivery, and patients' characteristics (age, number, and sex), in addition to bone marrow aspirate characteristics (nature, culture duration, sorting markers, culture passage, cellular dose, and activating agents) and defect characteristics (pathology, lesion size, and site).

### 2.4. Level of Evidence

The level-of-evidence rating introduced in the American Volume of the Journal of Bone and Joint Surgery in 2003 was used for assessment of all included studies [21].

### 2.5. Risk of Bias in Included Studies

The methodological bias in 4 randomized controlled trials was assessed by the Cochrane Collaboration's assessment tool for risk of bias from the Cochrane Handbook for Systematic Reviews. The following items were assessed as “low risk,” “high risk,” or “unclear risk” of bias: (1) random sequence generation, (2) allocation concealment, (3) blinding of participants and personnel, (4) blinding of outcome assessment, (5) incomplete outcome data, and (6) selective reporting addressed.

### 2.6. Quality Methodological Analysis [Modified Coleman Methodology Score (MCMS)]

Two authors independently reviewed and scored each study according to methodological criteria. The Coleman methodology score has been modified by Kon et al. [22] including the previously established ten criteria, giving a total score between 0 and 100. The two parts of MCMS grade cartilage-related studies based on ten criteria: part A—study size, mean follow-up, number of different surgical procedures, type of study, description of surgical procedure, postoperative rehabilitation, inclusion subjects' MRI outcome, and inclusion subjects' histological outcome, and part B—outcome criteria, procedure for assessing clinical outcomes, and description of subject selection process. A score of 100 indicates that the study largely avoids confounding factors and biases. The subsections that make up the Coleman methodology score are based on the subsections of the CONSORT statement (for randomized controlled trials) but are modified to allow for other trial designs.

### 2.7. Meta-Analysis

Standard meta-analytic methods were used to combine the results of all studies that provided sufficient data to obtain overall effect size estimates and the corresponding forest plots. Cochran's *Q* statistic was used to assess heterogeneity of the studies, and publication bias was assessed using funnel plots and fail-save analyses. All calculations were carried out using RevMan 5.3 software.

### 2.8. Statistical Methods

GraphPad Prism (version 5.0.0) was used to analyze the data. Descriptive statistics such as mean, standard deviation, range, median, and interquartile range were reported. The Pearson correlation coefficient was used for normally distributed data. Mean differences with their corresponding 95% confidence intervals (CIs) were generated for continuous outcome data (Visual Analog Scale for pain, International Knee Documentation Committee Function, Tegner Activity Scale, and Lysholm Knee Score), and *I*^2^ values were calculated to estimate the heterogeneity among the included studies. In the presence of homogeneity (*I*^2^ < 50%), the fixed effects model was used to estimate the overall effects. If there was significant heterogeneity among included studies, the random effects model was used. The meta-analysis was undertaken using RevMan 5.3 software.

## 3. Results

### 3.1. Search Result and Study Selection

Using the previously mentioned keywords, 518 relevant citations were obtained from the 3 online databases (PubMed, MEDLINE via Ovid, and Web of Science). The selection process ended up with thirty-three clinical trials which used BM-MSCs for knee cartilage lesions' repair. These 33 studies included 4 randomized controlled trials, 11 cases series, 7 case reports in addition to 7 observational cohort studies, and 2 studies in each category of pilot studies and phase I clinical trials.

### 3.2. Participants' Characteristics

724 patients were enrolled in the 33 clinical trials. 298 out of 724 were males. The mean age of all the included patients in 33 trials was 44.2 years ranging from 13 to 80 years. Most of the trials recruited patients with knee osteoarthritis with a severity grade of 1-4 on the K-L scale or OA grade system. OA and K-L grades 1-2 are considered as early OA; grades 3-4 as advanced OA. 12 studies and other 18 studies investigated the healing effect of BM-MSCs on early OA and advanced OA, respectively. The defect size was mentioned in nineteen studies with a wide range from 1.4 cm^**2**^ to 10 cm^**2**^. Regarding the defect site, it was variable including the femoral condyle, patella, tibia plateau, and trochlea. Most patients had a previous debridement, arthroscopy, and microfracture before receiving BM-MSC implantation. The duration of follow-up was stated clearly in thirty-one studies; the shortest period was three months in case reports done by Centeno et al. [23], and the mean of the longest follow-up duration was 75 months in the cases series performed by Wakitani et al. [24] (Table 1).

### 3.3. Intervention Characteristics

**Figure 2:**
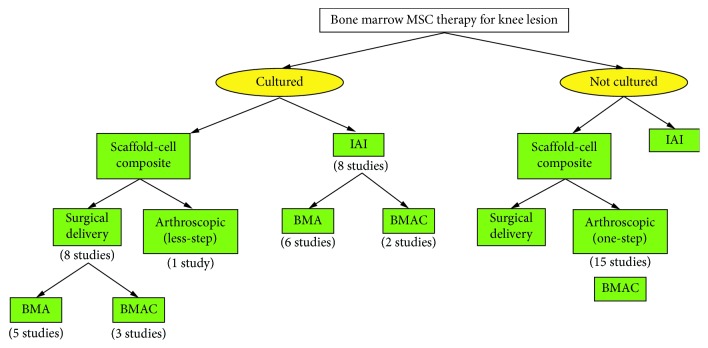
Classification of the included study-based preparation of BM-MSCs and delivery method.

All included studies utilized BM-MSC injection as an adjunct for knee cartilage repair. However, procedures used in the clinical trials were variable according to two parameters: the first parameter was the nature of BM-MSCs used, either cultured or noncultured, and the second one was the method of delivery (Figure 2). 368 patients in 16 studies received cultured BM-MSCs while another 16 studies used noncultured BM-MSCs for 355 patients. Centeno et al. delivered both cultured and noncultured BM-MSCs to their patients [23]. 15 out of 16 studies that used cultured BM-MSCs mentioned the details about the culture duration and passages. The mean duration of culturing was 9.5 days, ranging from 7 to 35 days. Moreover, most of the trials collected the cells from early passages. The mean number of passages was 1.6 passages with 1p to 5p as the range. The mean quantity of bone marrow aspirate harvested from the patients was 47 ml. Kasemkijwattana et al. [25] and Wakitani et al. [24] extracted only 10 ml. However, Kim et al. reported bone marrow aspiration of 120 ml. In studies using BMAC, the total amount of aspirate was concentrated down to 3-4 ml [26].

Seven studies implanted less than 10 million BM-MSCs. However, more than 20 million cells were delivered in 6 studies. Some did not specify the number of cells delivered. Fourteen studies (42%) reported MSC marker sorting prior to cellular delivery. CD90, CD29, CD44, and CD105 were used to detect MSCs while CD34, CD14, and HLA-DR were markers to eliminate the other cellular content. According to the method of cellular delivery, the studies were distributed among intra-articular injection (9 studies), surgical technique (9 studies), and arthroscopic one-step implantation (15 studies) (Tables 2 and 3).

### 3.4. Outcome Assessment Characteristics

There was a difference in tools used for outcome assessment on either patient-reported outcomes or measures assessed by the physicians. The most commonly used pain assessment tool was the Visual Analog Scale (VAS) for Pain (13 studies), followed by KOOS Pain (5 studies) and WOMAC pain (4 studies). In terms of function assessment, the International Knee Documentation Committee (IKDC) Score was commonly reported in eight studies, followed by Lysholm Score, Tegner Activity Scale, and KOOS Function in seven, six, and three studies, respectively. Most of the physicians used radiological assessment and 2nd look arthroscopy as reliable measures for therapy efficacy.

### 3.5. Level of Evidence

According to the level of evidence of the included studies, most of the studies were of level IV (21 studies), level I (four studies), level II (five studies), and level III (2 studies) of evidence (Table 4).

### 3.6. Quality Methodological Analysis

**Figure 3:**
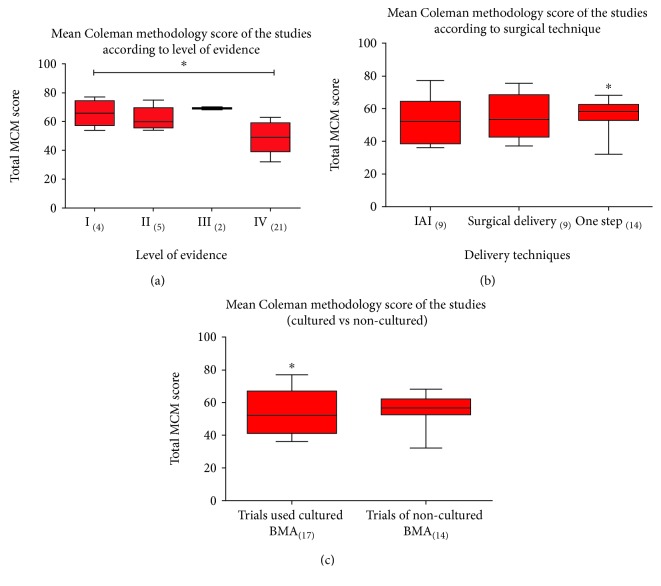
(a) Box plot showing the median, quartiles, and extreme values of the modified Coleman methodology score (CMS) for each level-of-evidence rating. (b) Box plot showing the median, quartiles, and extreme values of the modified Coleman methodology score (CMS) for each type of therapy. (c) Box plot showing the median, quartiles, and extreme values of the modified Coleman methodology score (CMS) for cell handling.

**Figure 4:**
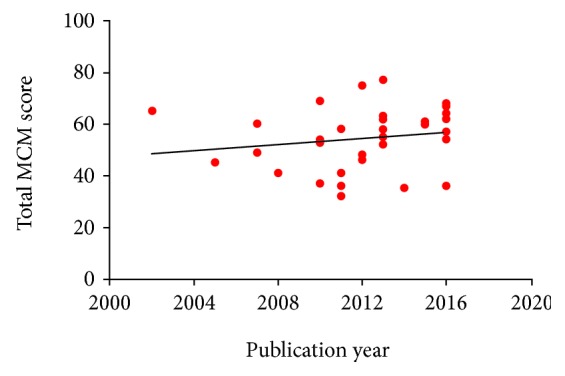
Modified Coleman methodology score (CMS) for studies reporting outcome after administration of BM-MSCs for cartilage repair plotted against publication year. There is a positive correlation (*r* = 0.16). The correlation was not statistically significant when unweighted (*P* = 0.3544).

There was a clear methodological defect in most studies with an average Coleman methodology score of 55 (25th-75th percentile, 45.5-63.5). There were low scores especially in the following: (1) type of study, (2) inclusion of histological outcome, (3) study size, and (4) mean duration of follow-up. The descriptive statistics for each criterion of the Coleman methodology score are described in table (Table 5). The distribution and correlation of the mean Coleman methodology score of the studies regarding level of evidence, type of therapy, and cellular centrifugation and culture are described in Table 4 and Figures 3(a), 3(b), and 3(c). To discover whether the methodological quality is trending up over the time or not, we correlated the total Coleman methodology score with year of publication (Figure 4). There is a positive correlation (*r* = 0.16). The correlation was not statistically significant (*P* = 0.3544).

### 3.7. Risk of Bias

**Figure 5:**
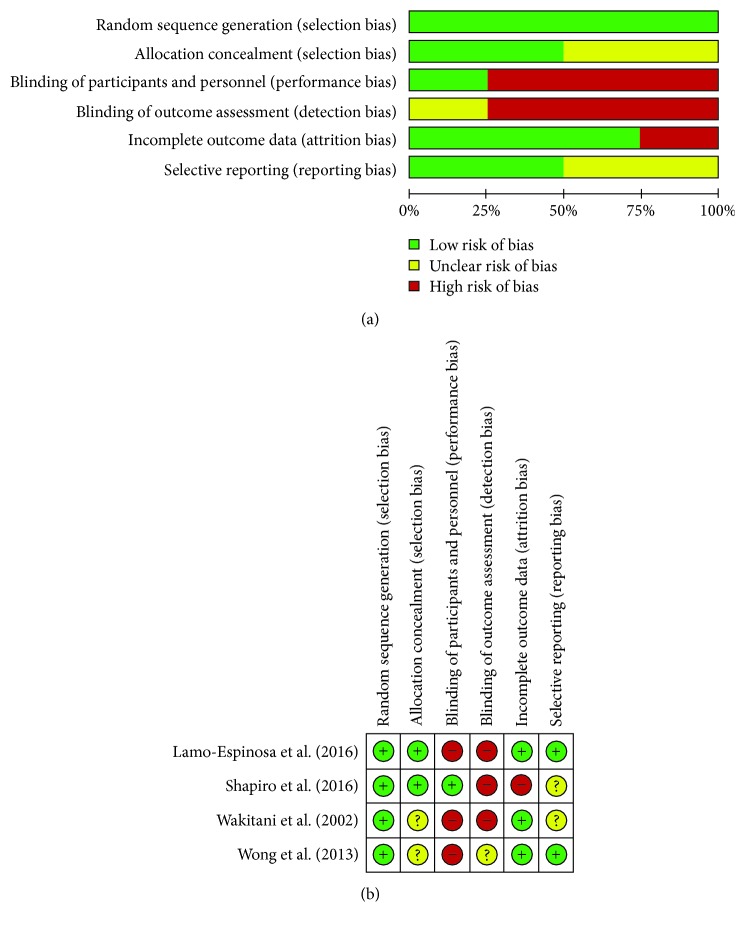
(a) Risk of bias graph: review authors' judgements about each risk of bias item presented as percentages across all included studies. (b) Risk of bias summary: review authors' judgements about each risk of bias item for each included study.

Regarding randomization and allocation, all 4 RCTs mentioned random sequence generation either briefly or in details, but allocation concealment was not clearly described in Wakitani et al. [27] and Wong et al. [28]. Almost all the 4 RCTs had a performance and detection bias. In terms of attrition bias, there was missing reported data in Wakitani et al. [27], Lamo-Espinosa et al. [29], and Wong et al. [28]. However, Shapiro missed some data. Review authors' judgements about each risk of bias item for each included study are described in Figures 5(a) & 5(b).

### 3.8. Meta-Analysis

#### 3.8.1. Visual Analog Scale for Pain (VAS Pain) after Receiving BMAC

**Figure 6:**
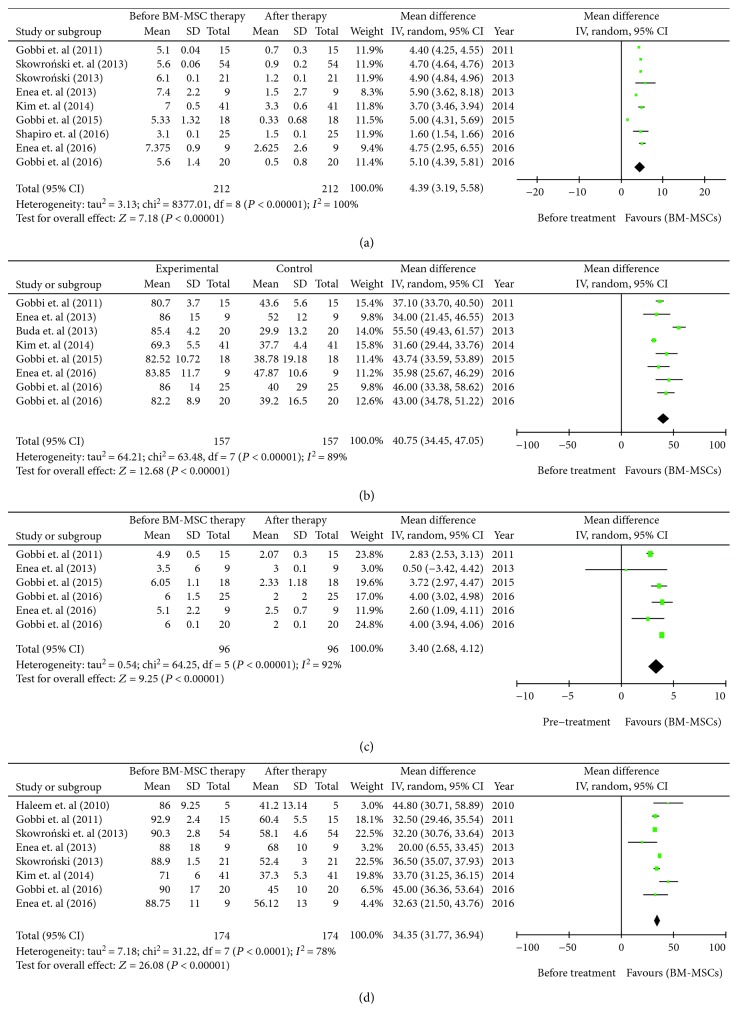
(a) Forest plot comparing the Visual Analog Scale for Pain (VAS Pain) before and after administration of noncultured bone marrow aspirate concentrate (BMAC). (b) Forest plot comparing the International Knee Documentation Committee (IKDC) function level before and after administration of noncultured bone marrow aspirate concentrate (BMAC). (c) Forest plot comparing Tegner Activity Scale function level before and administration of noncultured bone marrow aspirate concentrate (BMAC). (d) Forest plot comparing the Lysholm Knee Score before and after administration of noncultured bone marrow aspirate concentrate (BMAC).

Nine studies [26, 30–37]—including 217 patients—reported the data of Visual Analog Scale for Pain before and after receiving the one-step technique of noncultured bone marrow aspirate concentrate (BMAC). There was a statistically significant heterogeneity in the studies (*I*^2^ = 100%, *P* < 0.00001). Using the random effects model, the outcome results revealed that VAS after the administration of BM-MSCs was significantly much better than before the therapy (mean difference = 4.39, 95% CI: 3.19 to 5.58, *Z* = 7.18 (*P* < 0.00001) (Figure 6(a)).

#### 3.8.2. International Knee Documentation Committee (IKDC) Function after Receiving BMAC

Eight studies [26, 30–34, 38, 39]—including 157 patients—reported the data of International Knee Documentation Committee (IKDC) Function before and after receiving noncultured bone marrow aspirate concentrate (BMAC) (one-step technique). There was a statistically significant heterogeneity in the studies (*I*^2^ = 89%; *P* < 0.00001). Using the random effects model, the outcome results revealed that IKDC Function after the administration of BM-MSCs was significantly much better than before the therapy (mean difference = 40.75, 95% CI: 34.45 to 47.05, *Z* = 12.68 (*P* < 0.00001) (Figure 6(b)).

#### 3.8.3. Tegner Activity Scale after Receiving BMAC

Six studies [26, 30–33, 39]—including 96 patients—provided their data of the Tegner Activity Scale before and after receiving noncultured bone marrow aspirate concentrate (BMAC) (one-step technique). There was a statistically significant heterogeneity in the studies (*I*^2^ = 92%; *P* < 0.00001). Using the random effects model, the pooled results indicate that BM-MSC therapy exhibited much better improvement in activity scale when compared to before therapy (mean difference = 3.40, 95% CI: 2.68 to 4.12, *Z* = 9.25 with associated *P* < 0.00001) (Figure 6(c)).

#### 3.8.4. Lysholm Knee Score after Receiving BMAC

Six studies [26, 30–33, 39]—including 96 patients—provided their data of the Lysholm Knee Score before and after receiving noncultured bone marrow aspirate concentrate (BMAC) (one-step technique). There was a statistically significant heterogeneity in the studies (*I*^2^ = 78%; *P* < 0.00001). Using the random effects model, the pooled results indicate that BM-MSC therapy exhibited much better improvement in activity scale when compared to before therapy (mean difference = 34.35, 95% CI: 31.77 to 36.94, *Z* = 26.08 with associated *P* < 0.00001) (Figure 6(d)).

## 4. Discussion

To the best of our knowledge, this is the largest meta-analysis and systematic review including 33 clinical trials with 724 patients comparing their pain severity and knee function before and after receiving BM-MSCs for knee cartilage regeneration. The most important finding of our meta-analysis is that the administration of noncultured bone marrow aspirate concentrate (BMAC) can significantly reduce pain and improve knee function when compared to the scores before the therapy in almost all the evaluated studies [26, 30–33, 39–43].

Numerous studies believed that the immediate use or injection of native and freshly isolated autologous bone marrow aspirate has its own advantages. The heterogeneous composition of aspirate will synergistically foster the cartilage regeneration. In addition to avoiding any risk of rejection and any concerns about the lack of standardized protocols for stem cell preparation [44], most of included studies have aspirated the whole amount of autologous BM-MSCs from one insertion site in the iliac bone. Wakitani et al. [27] and Centeno et al. [23] aspirated half of the amount from the anterior superior iliac spine (ASIS) and the other half from the posterior superior iliac spine (PSIS). However, Adachi et al. [45] aspirate BM-MSCs from the tibial bone, while Wakitani et al. (3 ml × 5 times), Buda et al. (5 ml × 12 times), Wakitani et al. [27] (10 ml × 2 times), and Gigante et al. [41] (30 ml x 2 times) aspirated the amount of cells in a small fraction manner from different sites. Buda et al. [38] explained that the main aim of this different source collection is to maximize the harvesting of the marrow stromal stem cells and to reduce dilution by peripheral blood. The fact that the cellular composition of bone marrow aspirate is heterogeneous with only 0.001% mesenchymal stem cells led many investigators to adopt culturing of BM-MSCs after harvesting them to generate an adequate amount of MSCs followed by positive selection using BM-MSC-specific surface markers to confirm the identity of the injected cells [46].

In 2008, Centeno et al. [23] reported, for the first time, that intra-articular injection of expanded autologous MSCs into the knees would effectively regenerate knee cartilage. Their case report showed promising improvement in VAS Pain score and range of motion besides an increase in cartilage and meniscus volume in the MRI scan. They concluded that the patient's clinical response might have been due to the dexamethasone injection administrated after BM-MSC transplant, although the injected dose of dexamethasone (10 ng/ml) was approximately one million times lower than those used clinically. In addition, Centeno et al. could not determine if the regenerative tissue was fibrocartilage or true hyaline. In 2013, Orozco et al. [47] overcame this issue by using MRI T2 mapping to define the nature of regenerative tissue. After testing the effectiveness of this novel procedure by Centeno et al., Davatchi et al. [48] demonstrated the safety of cultured BM-MSC injection without any complications and reported encouraging outcomes of the preliminary study of four patients complaining about moderate to severe bilateral knee OA in the form of improvement in pain and function. More satisfactory results of intra-articular injection of BM-MSCs were shown by Emadedin et al. [49], in 2012, in six patients with OA. The comparison between MRI scans at the baseline and at 6 months postinjection displayed an increase in cartilage thickness which extended the regenerated tissue over the subchondral bone in 50% of patients, in addition to a considerable decrease in size of edematous patches that may be explained by the anti-inflammatory effect of MSCs. Based on these results, they recommended that BM-MSC injection would be effective for 6 months and a second injection may be required after. Emadedin et al. [49] claimed that their trial's outcome was much better than that of Davatchi et al. [48] and it could be attributed to the amount of cells injected.

The first randomized controlled trial was performed in 2013 by Wong et al. [28] and included 56 patients assigned to two groups. The cell-recipient group received cultured BM-MSCs and hyaluronic acid injection 3 weeks after. These patients showed significant improvement in either clinical scores or MOCART scores when compared to those who only received placebo and hyaluronic acid injection. Moreover, complete cartilage coverage has been reported in nine patients in the cell-recipient group. Furthermore, in advanced grade of knee OA, Soler et al. [50] obtained excellent clinical and quantitative MRI outcome measures with no adverse events after intra-articular injection of 40 × 10^6^ of autologous expanded BM-MSCs. Mehrabani et al. [51] also demonstrated satisfactory outcomes at 12 months after intra-articular injection of around 36 million BM-MSCs. They reported significant improvement on VAS Pain score and on functional status of the knee. In addition, regenerated cartilage extended over the subchondral bone as shown after 12 weeks in their posttransplantation MRI results. Lamo-Espinosa et al. [29] concluded that single intra-articular injection of 100 × 10^6^ in vitro expanded autologous BM-MSCs together with hyaluronic acid is a safe and feasible procedure that would result into a clinical and functional improvement for knee OA.

On the other hand, few studies have investigated the efficacy of allogenic BM-MSCs. Vangsness et al. [52] designed a randomized, double-blinded controlled trial to investigate the safety and clinical outcomes of intra-articular injection of cultured, allogeneic BM-MSCs from unrelated donors and not even matched to human leukocyte antigens of the recipient. They assigned three groups of patients with 3 different cellular doses: group A (18 patients) received 50 × 10^6^ cells; group B (18 patients) received 150 × 10^6^ cells; both groups received 2 ml of hylanurate Na, albumin, and plasmalyate A; and the control group also received them without a cellular component after 10 days following partial medial meniscectomy. The patients showed improvement in their Lysholm score and VAS Pain score compared to baseline values in all groups, and the overall group comparison was significant at 2 years of follow-up.

One of the key issues to maintain the efficacy, safety, and stemness of BM-MSCs cultured ex vivo is the nature and characteristics of the culture conditions. Most of the included studies, involving cultured BM-MSCs, used a medium supplemented with bovine serum cellular expansion (fetal bovine serum (FBS) [25, 28, 43, 47, 53, 54], fetal calf serum (FCS) [24, 27], or HyClone bovine serum [49, 51]). Using bovine serum in the culture medium has several disadvantages. First, it may be responsible for high variability, within the same study and even between passages. Secondly, it increases the risk of xenogeneic immune reactions and animal pathogen transmission [55, 56] . Intuitively, few included studies shifted to using human supplements such as patient's serum [45, 57, 58], or platelet lysate [23, 29] as an alternative to bovine serum. It has been demonstrated that the human supplements are effective and safe in MSC proliferation and maintenance of their phenotype and functionality [59, 60].

Despite the fact that early results of randomized controlled trial [35] showed significant improvement in the activity level of BMAC patients compared with baseline, many following studies assumed that scaffolds may be required for the regeneration of cartilage and could act as a cell carrier. Moreover, some scaffolds such as platelet-rich fibrin glue (PR-FG) have the advantage of being biodegradable and autologous and containing chondrogenesis-induced growth factors with sustained release. In addition, the ability of MSCs to differentiate and adhere to scaffolds have been investigated and proven [2, 17, 61]. In 2002, Wakitani et al. [27] presented a surgical transplantation technique for implanting BM-MSCs on collagen gel scaffold covered with periosteum flap in 12 osteoarthritic knees after undergoing high tibial osteotomy (HTO), compared to patients who underwent HTO alone. After a short-term follow-up, there was a significant improvement on histological and arthroscopic scores in the BM-MSC-implanted group. However, there was no difference in clinical improvement between the two groups. Wakitani et al. [27] have demonstrated that collagen gel can be used as delivery vehicles for chondrocytes. Wakitani et al. [24] presented their results after the treatment of nine cartilage defects in patellofemoral joints in five knees of three patients. They used BM-MSCs on collagen gel covered with periosteum in one case and synovium in the other two cases. Clinical improvement was reported at 7 months for case 1 who returned to work after 12 months, case 2 at 21 months, and case 3 at 8 months. In the same year, Kuroda et al. [57] reported the results of the implantation of autologous BM-MSCs within a focal cartilage defect of critical size (2-10 cm^2^) of the weight-bearing area of the medial femoral condyle in a 31-year-old male judo player through impeding into the collagen type I gel cell composite and covered with a periosteal flap from the anterior surface of tibia. Their follow-up showed clinical improvement, and the patient returned to his previous activity 1 year after surgery. In 2005, Adachi et al. [45] presented a case report of a 21-year-old man radiologically diagnosed with a large osteochondral knee defect as a complication of septic arthritis. This patient was surgically treated with cultured BMSCs on hydroxyapatite ceramic scaffold. The biopsy from these regenerated tissue showed cartilage and bone regeneration, with deficiency in proteoglycan content. Haleem et al. [43] demonstrated great promising outcome in five patients with transplantation of autologous cultured bone marrow MSCs in platelet-rich fibrin glue (PR-FG) scaffold in treatment of full-thickness cartilage defect, particularly large-sized defects (>4 cm^2^). They elucidated that PR-FG successfully fixed the cultured cells within defects and provided them a suitable environment to produce a hyaline-like cartilaginous matrix. MRI showed complete defect filling with smooth surface in three patients. However, incomplete regeneration was detected in two patients. In 2011, Kasemkijwattana et al. [25] used a 3D collagen scaffold seeded with autologous bone marrow MSCs, fixed with fibrin glue in treatment of two cases which had grade III-IV ICRS cartilage lesions, and after 30-31 months of follow-up, they reported excellent clinical improvement and the arthroscopic assessment demonstrated good defect fill and incorporation to the adjacent cartilage.

More recently, most of the clinical trials performed cellular implantation with a less invasive procedure to preserve the joint surface and encourage earlier return to full activity. Many investigators established the “one-step technique” of harvesting and implanting BM-MSCs in the same setting. Investigators who use this technique (involving the transfer of the entire bone marrow cellular pool into the site of the lesion in a single setting) assume that it preserves the regenerative potential of cells without the need of a laboratory phase and allows BMAC transplantation to be performed in “one step” instead of the two required for ACI [22, 38, 40]. Buda et al. [38] investigated the validity of the one-step technique to be a part of the cartilage repair paradigm through a case series of twenty patients with osteochondral lesions and associated other morbidities in the knee. All associated morbidities were repaired, and interestingly, biopsies showed cartilaginous tissue mainly type II and proteoglycan-rich matrix. Another group [62] reported positive results after injection of noncultured BMAC in 25 patients after debridement and compared them to 25 patients with debridement alone. During the follow-up, they observed an improvement in clinical symptoms, with shortening of length of hospital stay and better quality of life after BMAC injection. Gigante et al. [41] designed a study for 5 patients with symptomatic chondral lesions of the knee to undergo arthroscopic microfracture and implant a collagen type I scaffold seeded with bone marrow concentrate in an attempt to augment the outcome of the AMIC technique. They reported nearly normal arthroscopic appearance, but hyaline cartilage-like tissue was found in only one case. One year later, the same group [42] performed a new arthroscopic one-step technique “covered microfracture and bone marrow concentrate” (CMBMC). They suggested that this technique can be performed during the diagnostic arthroscopy and reported the ability of CMBMC to regenerate hyaline-like cartilage in lesions larger than 2 cm^2^.

The relation between the patient's age and viability of BM-MSCs and the consecutive outcomes of their transplantation for cartilage regeneration are still controversial. However, the degenerative process in patients with osteoarthritis resulted from deficiency of MSCs as the number and proliferative capacity of subchondral BM-MSCs decrease with age and advanced stage of OA [63, 64]. Some researchers are still able to harvest a sufficient amount of BM-MSCs in older patients with advanced osteoarthritis [65, 66]. Haleem et al. [43] concluded that age might have influenced the results of his scaffold-cell composite procedure for cartilage repair because the qualitative and quantitative difference in the metabolic activity of cells in the repair tissue is age-dependent [67]. Wong et al. in 2013 also documented that both their treatment arms achieved improvement in clinical scores, but after adjustment for age, baseline scores, and time of evaluation, the intra-articular injected group with MSCs showed significantly better scores than did the control group. On the other side of this opinion, Buda et al. [38] reported that age, gender, and cartilage lesion size did not affect the results of the one-step technique. Nejadnik et al. [54] designed an observational cohort study to compare between cartilage repair by using chondrocytes (36 patients) and by using BMMSCs (36 patients). Clinical outcomes had not been significantly affected by patients' age in the BMMSC group, while the patients younger than 45 years scored significantly better than patients older than 45 years in the ACI group. Gobbi et al. [32, 33, 39] reported complete coverage of lesions seen on magnetic resonance imaging with hyaline-like cartilage in 80% of patients. They presented with normal to nearly normal tissues (hyaline cartilage–like tissues) on histological biopsy performed at second-look arthroscopy, in addition to significant short-term improvement on pain and function assessment in patients younger than 45 years and with single and smaller lesion.

## 5. Recommendations and Guidelines for Future Trials

**Figure 7:**
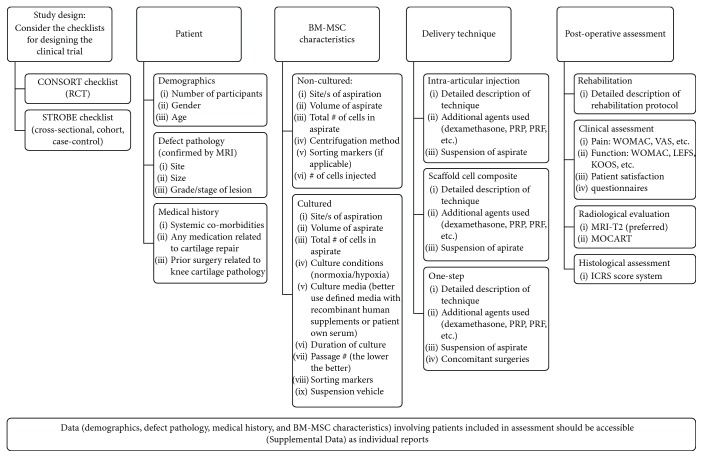
Guideline chart for the required data to be reported in future clinical trials.

Our systematic review aims to institute a more consistent and coherent guideline for reporting the minimum required information within the clinical studies evaluating the use of BM-MSCs in knee cartilage repair. Apart from that these guidelines would assist the investigators in presenting their trials' results, it would also guide the reviewers and the editors in critically appraising the BM-MSC studies in accord of the level of evidence, the validity, and the possibility of repeatability and reproducibility. There is a need that the orthopedic research community would collaboratively edit and participate in establishing some standard criteria for BM-MSC method reporting. A set of guidelines for the necessary information should be specified in any upcoming clinical trial investigating the safety and the effectiveness of BM-MSCs in knee cartilage repair (Figure 7).

### 5.1. Study Design

Of note, the aim of this section of our review was not only to recommend which minimum data should be reported, but rather to provide a framework for how the BM-MSC clinical study should be designed and stated. The STROBE [68] and CONSORT [69] checklists' items must be followed and reported by observational and randomized controlled trials, respectively.

### 5.2. Patients' Characteristics

Most of clinical studies in this field presented the basic demographic information about the included patients. However, additional data should be regularly addressed as well. First, the pathological and clinical characteristics of their cartilage defect should be fully noted in terms of the defect site, size, and grading system, in addition to the diagnosis approach on whether the clinical diagnosis of the defect was confirmed by magnetic reasoning imaging (MRI) or not. Secondly, it is necessary to note the medical history of the participants (e.g., systemic comorbidities, any previous medications related to cartilage repair, or any prior surgical procedures that had been performed either related or unrelated to the knee).

### 5.3. BM-MSC Characteristics

Some information is common between cultured and noncultured cellular aspirate and should be reported such as the site and volume of aspiration. In addition, total number of cells within the aspirate should be counted before the injection and clearly stated. In the case of using noncultured BM-MSCs, the technical method used for cell centrifugation and cellular surface markers for either positive or negative sorting should be added to the information stem. On the other hand, some other details about the cultured BM-MSCs would be importantly required. It is recommended to report the culture conditions to which the BM-MSCs have been exposed, the type and contents of culture media, and the culture duration as well as the number of passages.

### 5.4. Delivery Technique

There is a wide consensus with the necessity of reporting the detailed description of delivery technique including the surgical procedure, the suspension used for cellular injection, and the clear nomination of any additional agents that have been combined with the cells such as dexamethasone, PRP, and PRF.

### 5.5. Postoperative

Importantly, future trials would attempt to adopt consistent and established protocols for the optimal assessment of their outcome. This would also be helpful to estimate the definite effect size on a larger scale. The full details of the postoperative rehabilitation protocol should be noted. The currently available literature and the heterogeneity of outcome tools are challenging issues. First, there is a large variation in the used outcome measures in either clinical, radiological, or histological assessment of the knee joint after BM-MSC implantation. The statistical analysis performed in the systematic review [70] concluded the most reliable used measures to track the lower-extremity pain and function. Most of the studies recommended the (1) Western Ontario and McMaster Universities Osteoarthritis Index (WOMAC) pain and (2) Visual Analog Scores (VAS) assessing pain during activity and walking for pain assessment. Regarding the lower-extremity function, it is recommended to use the Lower Extremity Function Scale (LEFS), Functional Assessment System (FAS) test, Stratford Battery, and Physical Activity Restrictions (PAR) test. From the radiology stem point, MRI would be a gold standard option to either confirm the diagnosis or follow the cartilage regeneration after BM-MSC injection, while applying the ICRS score system on the biopsy harvested in the 2nd arthroscopy look would be an optimal method to examine the histology of the neotissues. The present study is not exempt from limitations. First, many of the included studies used BM-MSCswith either other surgical procedure or additional factors. Therefore, this may limit the accurate evaluation of BM-MSCs as an isolated therapy. Secondly, the variability and heterogeneity in outcome assessment tools lessen the ability to determine the effect size across the included studies.

The interest in the clinical use of MSCs for the management of knee OA has recently grown. However, the optimal dose and source of cells, as well as the use of coadjuvants, are not yet established. Large randomized controlled studies have enormous numbers of patients. In addition, longer periods for clinical, radiological, and histological follow-up are required for a much accurate investigation of MSC efficacy. Therefore, adopting a standard approach and adding the nuances that are ambivalent are crucial for future practice.

## Figures and Tables

**Table 1:** 

Characteristics	No. of studies	References
*Follow-up duration*		
0-6 months	2	[23, 35]
7-12 months	11	[28, 29, 34, 41, 42, 44–46, 48, 55]
13-24 months	8	[30, 32, 41, 47, 50–52, 62]
Up to 36 months	3	[25, 26, 38]
Over 3 years	6	[31, 33, 35–37, 39]
*Cellular dose*		
Less than 10 million	7	[24, 27, 36, 41, 45, 52, 55]
10-20 million	5	[27, 29, 50, 51, 56]
More than 20 million	6	[23, 29, 44, 46–48]

**Table 2:** 

Carriers used for implantation	No. of studies	References
*Scaffold material used in surgical delivery*		
Collagen	7	[24, 25, 27, 51, 52, 55, 56]
Ascorbic acid sheet	2	[51, 52]
Fibrin glue	1	[50]
Interconnected porous hydroxyapatite ceramic (IP-CHA)	1	[41]
*Scaffold material used in one-step arthroscopic technique*		
PGA/HA	1	[30]
collagen	6	[26, 32, 36, 37, 42, 62]
HA membrane	1	[33]
HA membrane with layer of PRF	1	[38]
HA membrane with fibrin glue	1	
HYAFF 11 nonwoven scaffold	2	[31, 39]
TruFit scaffold	1	
*Suspension used intra-articular injection*		
PBS	1	[23]
PBS+albumin	1	[45]
Autologous serum	1	[56]
Autologous platelet-poor bone marrow plasma	1	[35]
Synesthetic serum	1	[46]
Ringer's lactate	1	[44]

**Table 3:** 

	No. of studies	References
Studies used cultured BM-MSCs		
*Type of the culture media*		
DMEM supplemented with 10% FBS	6	[25, 28, 44, 50–52]
DMEM supplemented with 15% FCS	2	[24, 27]
*α*-MEM supplemented 10% HyClone bovine serum	2	[46, 48]
(DMEM) supplemented with 15% patient's serum	1	[41]
*α*-MEM+15% patient's serum	2	[55, 56]
(DMEM)+10% PL	1	[23]
*α*-MEM+5% platelet lysate+1 ng/ml bFGF	1	[29]

Studies used noncultured BM-MSCs		
*Centrifugation method*		
BMAC Harvest Smart PreP2 System	5	[31–34, 39]
MarrowStim Concentration Kit	5	[26, 30, 36, 37, 62]
Magellan Autologous System (Arteriocyte)	2	[35]
1000 g for 30 minutes	1	[25]
1000 g for 10 minutes	1	[23]
1500 g for 20 minutes	2	[46, 48]
3000 rpm for 3 minutes	1	[52]
3200 rpm for 15 minutes	1	[42]
3500 rpm for 6 minutes	1	

**Table 4:** Mean Coleman methodology score of the studies according to the level of evidence, type of therapy, and cell preparation.

	No. of studies	Mean Coleman methodology score (range)
*Level of evidence*		
I	4	66.25 (54-77)
II	5	62 (54-75)
III	2	68.5 (68-69)
IV	21	49 (32-63)
*Type of therapy*		
Intra-articular injection	9	52.2 (36-77)
One step	14	55.4 (32-68)
Surgical delivery	9	55.1 (37-75)
*Cell culture*		
Cultured	17	53.6 (36-77)
Noncultured	14	55.4 (32-68)
*Cellular centrifugation*		
BMAC	18	55.2 (35-69)
BMA	13	53.3 (32-77)

**Table 5:** Modified Coleman methodology score for studies using BMAC for knee cartilage repair.

Section score (maximum score)	Mean	Standard deviation	Range	Median	25th to 75th percentile
*Part A*					
Study size (10)	2.81	3.37	0-10	0	(0-5.5)
Mean duration of follow-up (10)	3.40	2.8	0-10	2	(2-5)
No. of surgical procedures (10)	9	2	4-10	10	(10-10)
Type of study (15)	4.2	5.8	0-15	0	(0-10)
Description of surgical procedure (5)	4.5	0.87	3-5	5	(4-5)
Description of postoperative rehabilitation (5)	2.45	2.41	0-5	2	(0-5)
Inclusion of MRI (10)	6.36	4.57	0-10	10	(0-10)
Inclusion of histological outcome (10)	3.3	3.88	0-10	0	(0-5)
*Part B*					
Outcome measures (5)	3.9	1.47	0-5	5	(3-5)
Outcome assessment (9)	5.56	2.09	2-9	5	(5-7)
Selection process (11)	9.3	2.6	3-11	11	(8-11)
Total part A (75)	36.2	10.03	14-54	37	(28-44)
Total part B (25)	18.7	4.99	5-25	19	(16-22)
Total score (100)	55	12.36	32-77	57	(45.5-63.5)
